# Clinical outcomes of combined anterior cruciate ligament and anterolateral ligament reconstruction: a systematic review and meta-analysis

**DOI:** 10.1186/s43019-021-00115-1

**Published:** 2021-09-23

**Authors:** Diego Ariel de Lima, Lana Lacerda de Lima, Nayara Gomes Reis de Souza, Rodrigo Amorim de Moraes Perez, Marcel Faraco Sobrado, Tales Mollica Guimarães, Camilo Partezani Helito

**Affiliations:** 1 UFERSA, Universidade Federal Rural do Semi-Árido, R. Francisco Mota, 572, Pres. Costa e Silva, Mossoró, RN CEP: 59625-900 Brazil; 2 Hospital Tarcísio Maia, Mossoró, Brazil; 3 Real Hospital Português, Recife, Brazil; 4 USP, Grupo de Joelho, Instituto de Ortopedia e Traumatologia, Hospital das Clínicas HCFMUSP, Faculdade de Medicina da Universidade de São Paulo, São Paulo, Brazil; 5 Hospital Sírio Libanês, São Paulo, Brazil

**Keywords:** Anterolateral ligament, Anterior cruciate ligament, Combined reconstruction, Isolated reconstruction, Clinical outcomes

## Abstract

**Objectives:**

To compare the clinical outcomes of isolated anterior cruciate ligament (ACL) reconstruction with combined reconstruction of the ACL and anterolateral ligament (ALL) of the knee.

**Methods:**

A search was conducted on the PubMed, Medline, Google Scholar, EMBASE, and Cochrane library databases, in line with the PRISMA protocol. The indexation terms used were “anterior cruciate ligament” OR “acl” AND “anterolateral ligament” AND “reconstruction.” Articles that compared patients submitted to combined ACL and ALL reconstruction with those submitted to isolated reconstruction of the ACL, with levels of evidence I, II, and III, were included. Studies with follow-up of less than 2 years and articles that did not use “anatomical” techniques for ALL reconstruction, such as extraarticular tenodesis, were excluded. A meta-analysis with R software was conducted, with a random effects model, presented as risk ratio (RR) or mean difference (MD), with a 95% confidence level (CI) and statistically significant at *p* < 0.05.

**Results:**

Ten articles were selected, with a total of 1495 patients, most of whom were men, of whom 674 submitted to ACL and ALL reconstruction and 821 to isolated ACL reconstruction. Combined ACL and ALL reconstruction exhibited a statistically significant advantage in residual pivot shift (RR 0.34, 95% CI 0.24–0.47, *I*^2^ = 0%, *p* < 0.01), rerupture rate (RR 0.34, 95% CI 0.19–0.62, *I*^2^ = 0%, *p* < 0.01), Lachman test (RR 0.59, 95% CI 0.40–0.86, *I*^2^ = 21%, *p* < 0.01), and postoperative Lysholm score (MD 2.28, CI 95% 0.75–3.81, *I*^2^ = 73%, *p* < 0.01).

**Conclusions:**

Combined ACL and ALL reconstruction obtained better postoperative clinical outcomes when compared with isolated ACL reconstruction, especially in reducing residual pivot shift and rerupture rate.

## Introduction

An anterior cruciate ligament (ACL) injury is very common, occurring mainly in sports [1, 2]. In the USA, more than 100,000 injuries are reported every year [3]. Although isolated ACL reconstruction is the standard treatment, a range of grafts and techniques are used [4, 5].

Despite the evolution of techniques, grafts, and implants, the rate of postoperative instability with isolated ACL reconstruction remains considerably high. The instability perceived by patients after ACL rupture is generally caused by pivot shift of the knee. It is estimated that up to 25% of ACL reconstructions evolve to residual pivot shift, revealing the inability of current isolated ACL reconstruction techniques to restore normal knee kinematics in many cases, especially rotatory stability [6, 7].

After thoroughly studying its anatomical and biomechanical properties, many authors believe that the anterolateral ligament (ALL) contributes to knee stability, by acting synergistically on the ACL, primarily in rotatory stability [3, 8–10]. These authors reported that a combined ACL and ALL injury may be responsible for some of the patients that do not evolve satisfactorily after isolated intraarticular ACL reconstruction, and recommend reconstructing the ALL in conjunction with the ACL to restore knee stability in specific cases [3, 11–14]. A large proportion of studies that compared combined ACL and ALL reconstruction displayed advantages in at least one parameter assessed, such as physical examination, subjective physical scales, and return-to-sport or rerupture rate.

A number of meta-analysis studies assessed extraarticular reconstructions as a large group and compared them with isolated ACL reconstructions, but few have evaluated only combined ACL and ALL reconstruction [15].

Thus, the aim of the present study is to systematically review and meta-analyze the clinical outcomes of isolated ACL reconstruction compared with combined ACL and ALL reconstruction, with a minimum of 24 months of follow-up, excluding other types of extraarticular reconstruction. Our hypothesis is that patients submitted to combined ACL and ALL reconstruction exhibit less residual laxity and rotatory instability and better clinical outcomes compared with those submitted to isolated ACL reconstruction.

## Materials and methods

In February 2021, two of the authors independently searched the PubMed, Medline, Google Scholar, EMBASE, and Cochrane library databases, with no date restrictions. The review was carried out according to PRISMA protocol recommendations [16].

The following indexing terms were used: “anterior cruciate ligament” OR “acl” AND “anterolateral ligament” AND “reconstruction.” The titles and abstracts were used to select articles that met the objective of study. Thus, only articles with a surgery protocol and follow-up of combined ACL and ALL reconstruction in their title or abstract were selected.

The articles selected were read in their entirety and their reference lists searched manually for additional relevant studies. Only complete versions of articles or those that had at least an abstract in English were accepted.

The inclusion criteria were articles with patients submitted to anatomical ALL combined with ipsilateral ACL reconstruction, either primary or revision, with levels of evidence I, II, and III. Study designs including randomized clinical trials (level I) and prospective or retrospective cohort studies (level II e III) were accepted. All level I evidence studies were included. Level II and III studies had the risk of bias assessed using the Newcastle–Ottawa Scale (NOS) [17]. The NOS was used to evaluate the methodological quality of evidence (MQOE) for each included study. This is a 9-point scale with 7–9 points representing very good MQOE, 5–6 points representing good MQOE, 4 points representing satisfactory MQOE, and 0–3 points representing unsatisfactory MQOE. Studies evaluated as very good and good MQOE were included.

Studies in which the patients were followed for less than 2 years, in which the research was purely biomechanical and anatomical, or which used any extraarticular technique other than ALL reconstruction were excluded.

### Statistical analysis

A meta-analysis of the data was carried out using the random effects model when the heterogeneity of the papers compared according to each parameter exceeded 50% and using the fixed effects model when the heterogeneity was less than 50%. Results were presented as risk ratio (RR) or mean difference (MD) with a 95% confidence interval (CI) and statistically significant at *p* < 0.05. Statistical analysis was conducted with R software, version R 4.0.3 GUI 1.73 for Mac OS X, meta package 4.15-1 [18]. Heterogeneity was assessed using *I*^2^ statistics, where an *I*^2^ value near 0% indicates nonheterogeneity between the studies, near 25% low heterogeneity, near 50% moderate heterogeneity, and near 75% high heterogeneity [19]. The following methods were used for analyses presented as risk ratio: Mantel-Haenszel method, DerSimonian-Laird estimator for *τ*^2^, Mantel-Haenszel estimator used to calculate *Q* and *τ*^2^ (such as RevMan 5) and continuity correction of 0.5 in studies with zero cell frequencies. For analyses presented as mean difference, the following methods were used: Inverse variance method, DerSimonian-Laird estimator for *τ*^2^ and Jackson’s method for confidence interval of *τ*^2^ and *τ*.

## Results

**Fig. 1:**
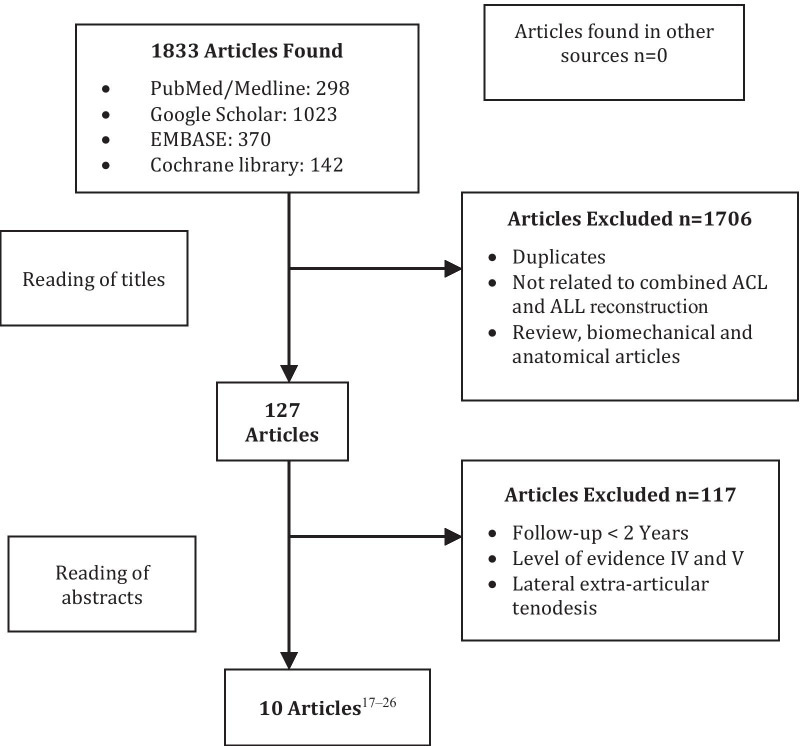
Flowchart of the articles selected

A total of 298 articles were found in PubMed/Medline, 1023 in Google Scholar, 370 in EMBASE, and 142 in Cochrane library. After articles simultaneously indexed in more than one database were excluded, 291 articles remained. Of these, 164 were excluded because they were purely biomechanical or anatomical and did not have the minimum follow-up. Of the remaining 117 articles, only 10 met the established inclusion criteria [20–29] (Fig. 1).

### Study characteristics

Of the ten studies, three were prospective randomized clinical trials (level of evidence I [20–22]), while the other two studies were prospective cohort studies (level of evidence II [23, 24]) and five retrospective studies (level of evidence III [25–29]). Of the ten articles selected [20–29], all used the ACL and ALL reconstruction techniques, and had the minimum 24-month follow-up (Table 1). All the studies compared their results with those of a control group consisting of isolated ACL reconstruction (Table 2)

**Table 1:** Articles selected: results of combined ACL and ALL reconstruction. *LOE* level of evidence, *NOS* Newcastle–Ottawa Scale

Study	LOE/NOS	*N*	Age	Indication	Technique	Preoperation	Postoperation	Preoperation	Postoperation	Return-to-sport rate
Goncharov et al. (2019) [23]	II/8	18		Professional sport activities or participation in competitions; age between 16 and 40 years	ACL: patellar ALL: hamstring	Lachman test: 18 Pivot shift: 18	24 months Lachman test: 0 Pivot shift: 0	IKDC score: 63.1 ± 4.8 Lysholm score: 72.6 ± 6.45	24 months IKDC score: 96.3 ± 1.8 Lysholm score: 97.4 ± 1.18	100%
Helito et al. (2019) [25]	III/6	30	27.0 ± 9.1	Laxity based on the modified Beighton scale [45] with a minimum value of 5–8	ACL: hamstring ALL: gracilis	Pivot shift: 30 Rolimeter arthrometer: 7.7 ± 1.3 mm Beighton scale: 6.1 ± 1.1	28.1 ± 4.2 months Pivot shift: 8 Rolimeter arthrometer: 1.5 ± 1.1 mm		28.1 ± 4.2 months IKDC score: 86.9 ± 9.3 Lysholm score: 88.3 ± 7.3 Rerupture: 1	
Ibrahim et al. (2017) [20]	I	53	26 (20–32)	At least two of the following criteria: grade 2/3 pivot shift; competitive sport; pivoting sport; chronic ACL injury; Segond fracture	ACL: hamstring ALL: gracilis	Pivot shift: 53 Lachman test: 53 KT-1000 arthrometer: 9.0 ± 3.0	27 months Pivot shift: 5 Lachman test: 4 KT-1000 arthrometer: 1.3 ± 0.2	Lysholm score: 75.0 ± 15.0 Tegner score: 6.0 ± 2.0	27 months Lysholm score: 98.0 ± 5.0 Tegner score: 8.0 ± 1.0	
Sonnery-Cottet et al. (2018) [26]	III/8	189	23.8 ± 6.8	At least two of the following criteria: grade 3 pivot shift; competitive sport; pivoting sport; chronic ACL injury; Segond fracture; lateral femoral notch sign; age up to 25 years	ACL: hamstring or patellar ALL: gracilis				36.6 ± 8.2 months Lysholm score: 93.7 Tegner score: 7.2	61.2%
Helito et al. (2018) [27]	III/7	33	33.1 ± 8.8	Simple ACL rupture more than 1 year before	ACL: hamstring ALL: gracilis	Pivot shift: 33 KT-1000 arthrometer: 8.39 ± 1.1	25 (24–28) months Pivot shift: 3 KT-1000 arthrometer: 1 (1–2) mm		25 (24–28) months IKDC score: 92.7 ± 5.9 Lysholm score: 95.4 ± 5.3 Rerupture: 0	
Sonnery-Cottet et al. (2017) [24]	II/7	221	21.8 ± 4.0	ACL rupture in young people + pivoting sport	ACL: hamstring ALL: gracilis	Side-to-side laxity: 7.5 ± 1.6	35.4 ± 8.4 months Side-to-side laxity: 0.5 ± 0.8	IKDC score: 57.2 ± 20.2	35.4 ± 8.4 months IKDC score: 81.8 ± 13.1 Lysholm score: 91.9 ± 10.2 Tegner score: 7.0 ± 2.0 Rerupture rate: 4.13%	68.8%
Yoon et al. (2020) [28]	III/6	18	32.9 ± 10.8	Revision ACL + grade 2 or 3 pivot shift	ACL and ALL: allograft	Anterior drawer: 17 Pivot shift: 18 Lachman test: 18 Side-to-side difference.: 7.7 ± 2.9	2 years Anterior drawer: 10 Pivot shift: 8 Lachman test: 12 Side-to-side difference.: 3.9 ± 3.0	IKDC score: 46.3 ± 11.2 Lysholm score: 51.6 ± 13.5 Tegner score: 2.9 ± 0.8	2 years IKDC score: 57.8 ± 15.7 Lysholm score: 58.7 ± 16.1 Tegner score: 4.0 ± 1.7 Rerupture: 2	
Hamido et al. (2020) [21]	I	50	24 (18–33)	ACL rupture in athletes + pivot shift	ACL: hamstring ALL: gracilis	Pivot shift: 50 KT-1000 arthrometer: 11.5 ± 0.8	60 (55–65) months Anterior drawer: 3 Pivot shift: 2 Lachman test: 2 KT-1000 arthrometer: 1.2 ± 0.7	Lysholm score: 72 ± 13.5 Tegner score: 6.4 ± 1.2	60 (55–65) months Lysholm score: 96 ± 5.0 Tegner score: 7.9 ± 0.8 Rerupture: 0	100%
Abdelrazek et al. (2019) [22]	I	20	24.9 ± 7.2	Chronic ACL rupture or grade 3 pivot shift	ACL: hamstring ALL: gracilis	Pivot shift: 20 Lachman test: 20	2 years Pivot shift: 2 Lachman test: 5 Internal tibial rotation angle side-to-side difference: 2.0 ± 1.17 KT-1000 arthrometer side-to-side difference: 1.1 ± 0.8			
Lee et al. (2019) [29]	III/6	42	26.8 ± 6	Revision ACL and age up to 45 years	ACL: anterior tibial allograft ALL: gracilis allograft	Pivot shift: 42 Lachman test: 42 KT-2000 arthrometer: 9.8 ± 1.7	Pivot shift: 4 Lachman test: 3 KT-2000 arthrometer: 1.9 ± 1.3	IKDC score: 68.7 ± 17.3 Lysholm score: 74.4 ± 16.1 Tegner score: 5.4 ± 0.8	IKDC score: 79.2 ± 18.8 Lysholm score: 88.5 ± 16.9 Tegner score: 6.7 ± 0.7	57.1%

**Table 2:** Articles selected: results of control groups (isolated ACL reconstruction)

Study	LOE/NOS	Control (*N*)	Age	Indication	Preoperation	Postoperation	Preoperation	Postoperation	Return-to-sport rate
Goncharov et al. (2019) [23]	II/8	30		Professional sport activities or participation in competitions; age between 16 and 40 years	Lachman test: 30 Pivot shift: 30	24 months Lachman test: 13 Pivot shift: 11	IKDC score: 73.4 ± 3.206 Lysholm score: 69.6 ± 3.51	24 months IKDC score: 90.3 ± 3.73 Lysholm score: 92.1 ± 3.935	66.7%
Helito et al. (2019) [25]	III/6	60	29.9 ± 8.1	Laxity based on the modified Beighton scale [45] with a minimum value of 5–8	Pivot shift: 60 Rolimeter arthrometer: 7.4 ± 1.2 mm Beighton scale: 5.8 ± 0.9	29.6 ± 6.2 months Pivot shift: 31 Rolimeter arthrometer: 2.3 ± 1.4 mm		29.6 ± 6.2 months IKDC score: 84.3 ± 9.8 Lysholm score: 86.3 ± 7.8 Rerupture: 13	
Ibrahim et al. (2017) [20]	I	50	26 (20–32)	At least two of the following criteria: grade 2/3 pivot shift; competitive sport; pivoting sport; chronic ACL injury; Segond fracture	Pivot shift: 50 Lachman test: 50 KT-1000 arthrometer: 8.1 ± 3.2	27 months Pivot shift: 6 Lachman test: 5 KT-1000 arthrometer: 1.8 ± 0.8	Lysholm score: 72.0 ± 13.5 Tegner score: 6.0 ± 2.0	27 months Lysholm score: 96.0 ± 3.5 Tegner score: 8.0 ± 1.0	
Sonnery-Cottet et al. (2018) [26]	III/8	194	30.9 ± 9.9	At least two of the following criteria: grade 3 pivot shift; competitive sport; pivoting sport; chronic ACL injury; Segond fracture; lateral femoral notch sign; age up to 25 years				39.2 ± 9.4 months Lysholm score: 93.0 Tegner score: 6.5	63.0%
Helito et al. (2018) [27]	III/7	68	33.9 ± 6.1	Simple ACL rupture more than 1 year before	Pivot shift: 68 KT-1000 arthrometer: 8.25 ± 1.1	26 (24–29) months Pivot shift: 24 KT-1000 arthrometer: 2 (1–2) mm		26 (24–29) months IKDC score: 87.1 ± 13 Lysholm score: 91 ± 2.3 Rerupture: 5	
Sonnery-Cottet et al. (2017) [24]	II/7	Patellar: 105 Hamstring: 176	Patellar: 22.1 ± 3.7 Hamstring: 23.5 ± 4.0	ACL rupture in young people + pivoting sport	Patellar: Side-to-side laxity: 7.6 ± 1.6 Hamstring: Side-to-side laxity: 7.4 ± 1.5	Patellar: 39.2 ± 8.8 months Side-to-side laxity: 0.6 ± 0.9 Hamstring: 41.6 ± 7.0 months Side-to-side laxity: 0.6 ± 1.0	Patellar: IKDC score: 56.5 ± 15.8 Hamstring: IKDC score: 59.4 ± 16.3	Patellar: 39.2 ± 8.8 months IKDC score: 86.8 ± 10.5 Lysholm score: 92.4 ± 8.6 Tegner score: 7.4 ± 2.1 Rerupture rate: 16.77% Hamstring: 41.6 ± 7.0 months IKDC score: 85.4 ± 10.4 Lysholm score: 91.3 ± 9.9 Tegner score: 6.6 ± 1.8 Rerupture rate: 10.77%	Patellar: 63.5% Hamstring: 59.9%
Yoon et al. (2020) [28]	III / 6	21	29.6 ± 10.2	Revision ACL + grade 2 or 3 pivot shift	Anterior drawer: 21 Pivot shift: 21 Lachman test: 21 Side-to-side difference: 8.0 ± 3.6	2 years Anterior drawer: 17 Pivot shift: 18 Lachman test: 21 Side-to-side difference: 5.9 ± 2.8	IKDC score: 46.8 ± 19.4 Lysholm score:48.4 ± 25.3 Tegner score: 3.5 ± 2.1	2 years IKDC score: 56.4 ± 20.7 Lysholm score:62.0 ± 21.3 Tegner score: 4.0 ± 2.7 Rerupture: 3	
Hamido et al. (2020) [21]	I	52	26 (18–40)	ACL rupture in athletes + pivot shift	Pivot shift: 52 KT-1000 arthrometer: 10.2 ± 0.8	60 (55–65) months Anterior drawer: 7 Pivot shift: 9 Lachman test: 8 KT-1000 arthrometer: 2.5 ± 0.7	Lysholm score: 74 ± 14.5 Tegner score: 6.9 ± 1.6	60 (55–65) months Lysholm score: 94 ± 4.5 Tegner score: 7.8 ± 1.4 Rerupture: 5	100%
Abdelrazek et al. (2019) [22]	I	20	26.6 ± 7.2	Chronic ACL rupture or grade 3 pivot shift	Pivot shift: 20 Lachman test: 20	2 years Pivot shift: 4 Lachman test: 4 Internal tibial rotation angle side-to-side difference: 2.0 ± 1.17 KT-1000 arthrometer side-to-side difference: 1.3 ± 1.3			
Lee et al. (2019) [29]	III/6	45	27.3 ± 7.6	Revision ACL and age up to 45 years	Pivot shift: 45 Lachman test: 45 KT-2000 arthrometer: 9.4 ± 1.4	Pivot shift: 20 Lachman test: 5 KT-2000 arthrometer: 2.2 ± 1.4	IKDC score: 67.1 ± 16.4 Lysholm score: 73.2 ± 15.6 Tegner score: 5.2 ± 1.1	IKDC score: 76.7 ± 17.2 Lysholm score: 85.1 ± 18.4 Tegner score: 6.5 ± 0.9	25.6%

*LOE* level of evidence, *NOS* Newcastle–Ottawa Scale

### Patients

The studies included 1495 patients, mostly men, aged between 20 and 30 years (674 submitted to ACL and ALL reconstruction and 821 controls), and the majority with injuries sustained playing professional or amateur sports. In the articles that specified which sport the patients played, soccer was the most common (51.7%).

### Indication for ACL and ALL reconstruction

Nine different indications were found as inclusion criteria for combined ACL and ALL reconstruction. The studies used at least one or a combination of these indications.

The most frequent was the presence of grade 2 or 3 pivot shift, with five studies [20–22, 26, 28], followed by participation in a competitive sport [20, 21, 23, 26] and chronic ACL injury [20, 22, 26, 27], both cited in four studies.

Four studies used age as an indication (between 16 and 40 years [23], young people [24], age up to 25 years [26] and age up to 45 years [29]), three used participation in pivoting sports [20, 24, 26], two used Segond fracture [20, 26], and two used revision ACLR [28, 29].

The rest were ligamentous laxity [25] and radiologic signs of lateral femoral notch [26].

### Clinical outcomes

The most widely used preoperative and postoperative clinical outcomes were pivot shift, rerupture rate, Lachman test, return-to-sport rate, IKDC score, Lysholm score, and Tegner score.

### Pivot shift

**Fig. 2:**
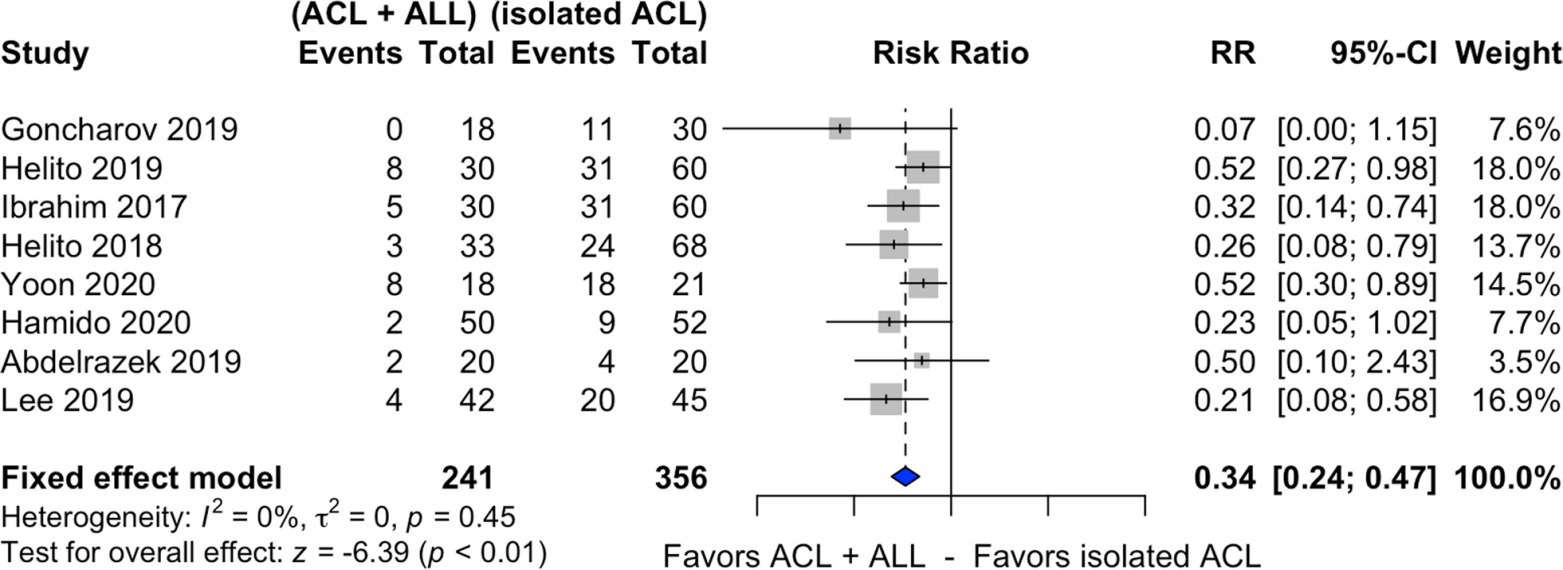
Forest plot of postoperative residual pivot shift of the combined ACL and ALL reconstruction and isolated ACL reconstruction groups

Eight studies assessed preoperative and postoperative pivot shift [20–23, 25, 27–29] (Fig. 2), with 241 patients submitted to combined ACL and ALL reconstruction and 356 to isolated ACL reconstruction. Among the patients submitted to the latter, 34.5% exhibited residual pivot shift. This rate declined to 13.2% for the combined ACL and ALL reconstruction.

Combined ACL and ALL reconstruction reduced the residual pivot shift rate by 66%, compared with the isolated ACL reconstruction (RR 0.34, 95% CI 0.24–0.47, *p* < 0.01). The *I*^2^ statistic indicated nonheterogeneity between the studies (*I*^2^ = 0%).

### Rerupture rate

**Fig. 3:**
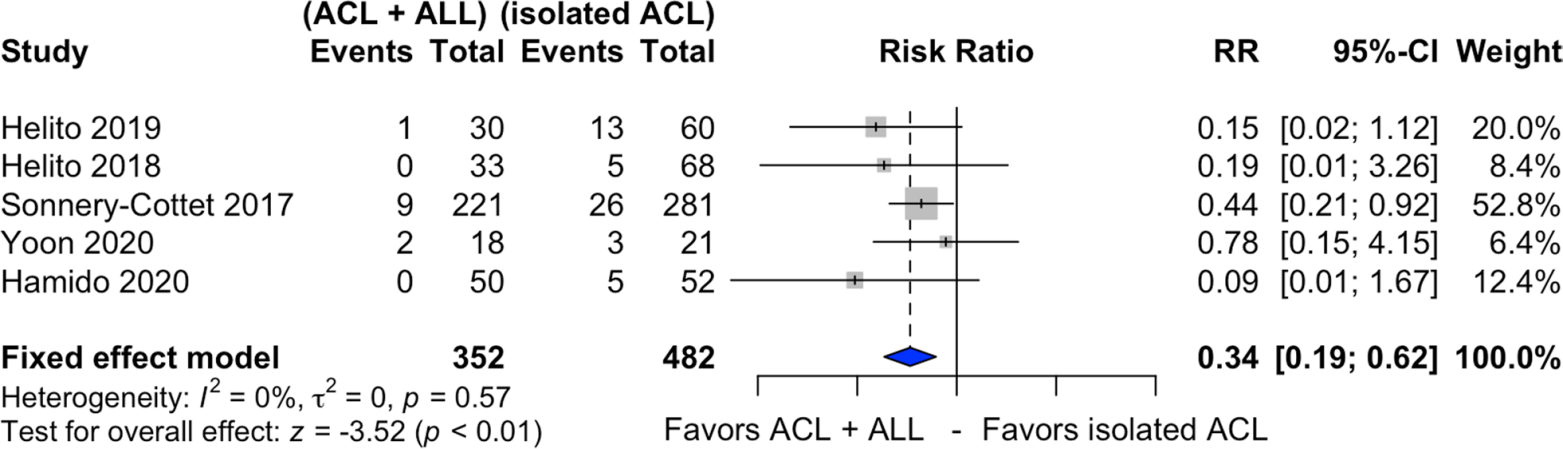
Forest plot of postoperative rerupture rate of the combined ACL and ALL reconstruction and isolated ACL reconstruction groups

Five studies assessed the postoperative graft rerupture rate [21, 24, 25, 27, 28] (Fig. 3), with 352 patients submitted to combined ACL and ALL reconstruction and 482 to isolated ACL reconstruction. Among patients submitted to the latter, the rerupture rate was 10.7%. In combined ACL and ALL reconstruction, this rate decreased to 3.4%.

Combined ACL and ALL reconstruction reduced the postoperative graft rerupture rate by 66%, compared with its isolated counterpart (RR 0.34, 95% CI 0.19–0.62, *p* < 0.01). The *I*^2^ statistic indicated nonheterogeneity between the studies (*I*^2^ = 0%).

### Lachman test

**Fig. 4:**
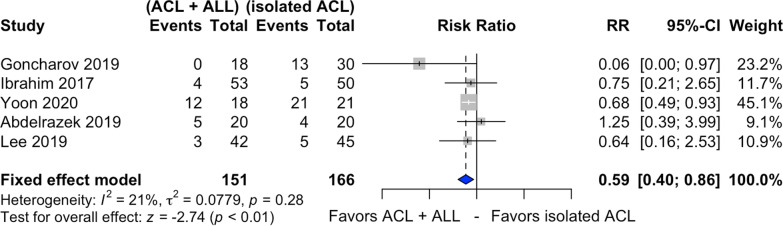
Forest plot of postoperative residual Lachman test of the combined ACL and ALL reconstruction and isolated ACL reconstruction groups

Five studies assessed the preoperative and postoperative Lachman test [20, 22, 23, 28, 29] (Fig. 4), with 151 patients submitted to combined ACL and ALL reconstruction and 166 to isolated ACL reconstruction. Among those submitted to the latter, 28.9% exhibited a positive postoperative residual Lachman test, declining to 15.8% for combined ACL and ALL reconstruction.

Combined ACL and ALL reconstruction decreased residual Lachman test by 41%, compared with its isolated counterpart (RR 0.59, 95% CI 0.40–0.86, *p* < 0.01). The inconsistency can be considered low (*I*^2^ = 21%).

### Return to sport rate

**Fig. 5:**
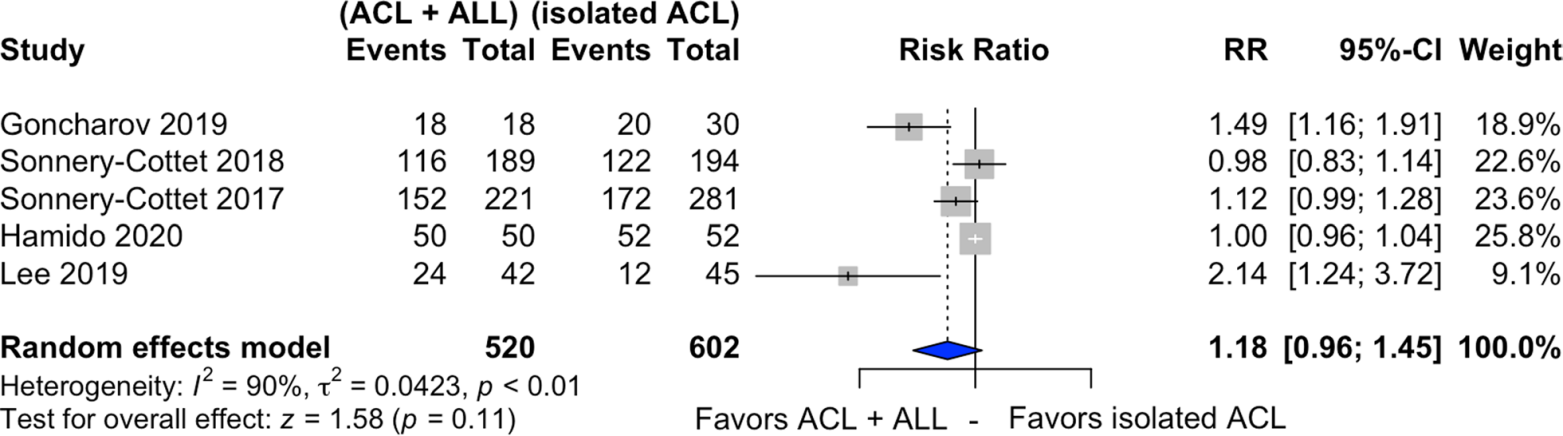
Forest plot of postoperative return-to-sport rate of the combined ACL and ALL reconstruction and isolated ACL reconstruction groups

Return to sport was assessed in five studies [21, 23, 24, 26, 29] (Fig. 5), with 520 patients submitted to combined ACL and ALL reconstruction and 602 to isolated ACL reconstruction. Among patients submitted to the latter, 62.7% returned to the sport after surgery. In the combined ACL and ALL reconstruction, this rate rose slightly to 69.2%.

Combined ACL and ALL reconstruction increased the return-to-sport rate by 18%, compared with simple reconstruction (RR = 1.18, 95% CI 0.96–1.45, *p* = 0.11). The *I*^2^ statistics indicated high heterogeneity between the studies (*I*^2^ = 90%).

### IKDC score

**Fig. 6:**
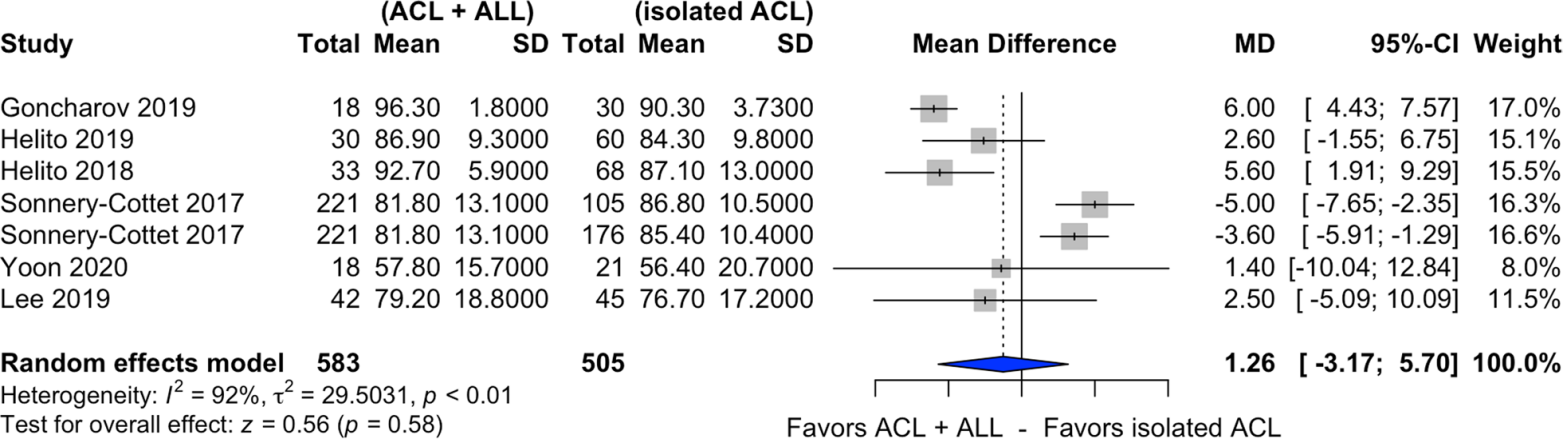
Forest plot of postoperative IKDC score of the combined ACL and ALL reconstruction and isolated ACL reconstruction groups

Six of the ten studies selected assessed postoperative IKDC score [23–25, 27–29] (Fig. 6). In relation to this score, there was a nonsignificant difference in favor of combined ACL and ALL reconstruction (MD 1.26, CI 95% 3.17–5.70, *I*^2^ = 92%, *p* = 0.58).

### Lysholm score

**Fig. 7:**
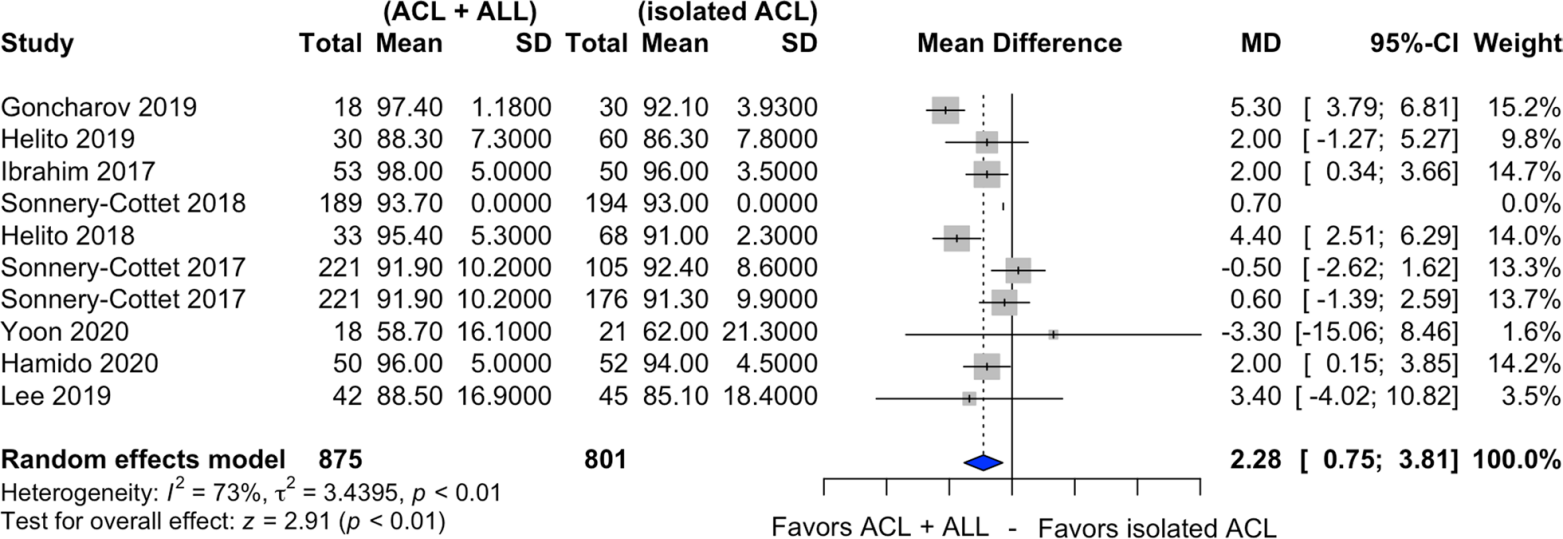
Forest plot of postoperative Lysholm score of the combined ACL and ALL reconstruction and isolated ACL reconstruction groups

Nine of the ten studies selected assessed postoperative Lysholm score [20, 21, 23–29] (Fig. 7). In relation to this score, there was a statistically significant difference in favor of combined ACL and ALL reconstruction (MD 2.28, CI 95% 0.75–3.81, *I*^2^ = 73%, *p* < 0.01).

### Tegner score

**Fig. 8:**
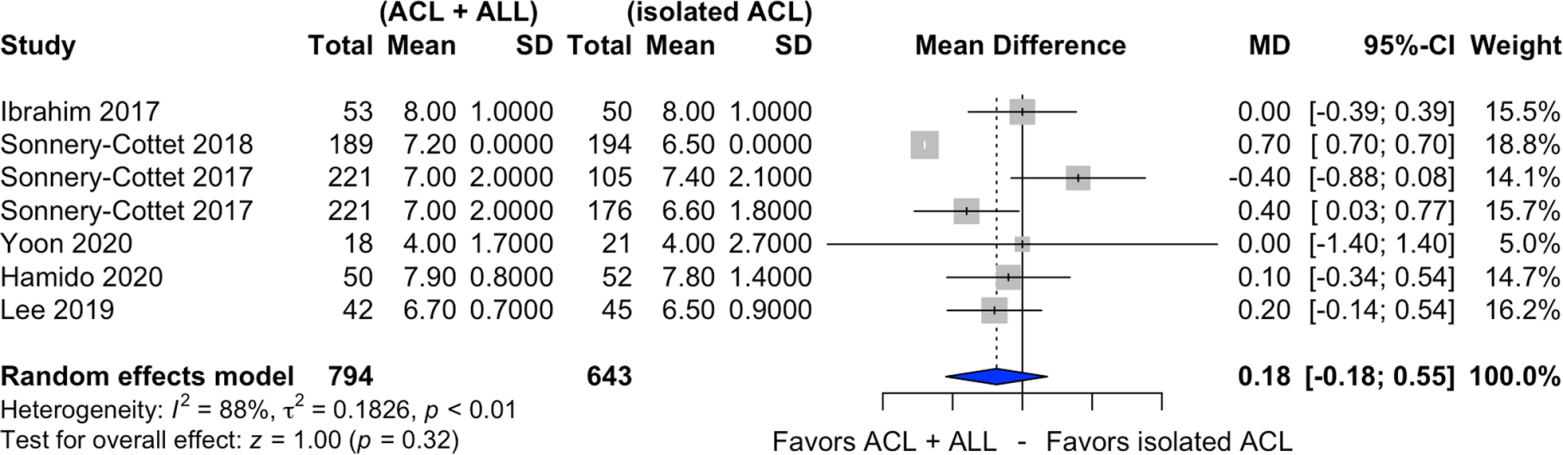
Forest plot of postoperative Tegner score of the combined ACL and ALL reconstruction and isolated ACL reconstruction groups

Six of the ten studies selected assessed postoperative Tegner score [20, 21, 24, 26, 28, 29] (Fig. 8). In relation to this score, there was a nonsignificant difference in favor of combined ACL and ALL reconstruction (MD 0.18, CI 95% −0.18 to 0.55, *I*^2^ = 88%, *p* < 0.01).

## Discussion

The main finding of the present meta-analysis was that combined ACL and ALL reconstruction exhibits a lower rerupture rate, better Lysholm score, lower residual pivot shift rate, and lower residual Lachman test positive rate compared with isolated ACL reconstruction.

Biomechanical studies demonstrated that the ALL exhibits an injury mechanism similar to that of the ACL, is an important stabilizer against anterolateral tibial rotation, and affects pivot shift in ACL failure [30–36]. Some authors believe that a combined ACL and ALL injury may account for a certain percentage of patients that do not evolve satisfactorily after isolated intraarticular ACL reconstruction and recommend combining it with ALL reconstruction to restore knee stability, especially for a carefully selected group of patients [3].

The long-term results of isolated ACL reconstruction are good in terms of restoring joint stability, enhancing symptoms, and returning to the activities practiced before the injury. However, 0.7–20% of the patients displayed recurring instability due to graft failure [37, 38] and the global revision rate was 8.4% [39], with a higher rate in at-risk populations. Webster and Feller [40] found a rerupture rate of 18% in patients younger than 18 years old and Larson et al. [41] 24.4% in those with hyperlaxity.

The main objective of combined ACL and ALL reconstruction is greater rotational control and prevention of ACL rerupture, given that the ALL divides the forces with the ACL, thereby avoiding overloading the latter [42, 43]. Thus, we can infer that the best indications for combined ACL and ALL reconstruction would be the clinical conditions that exhibit rotatory instability and greater risk of rerupture [42, 43]. Although there is no absolute indication for combined ACL and ALL reconstruction, recent consensus includes patients with high pivot shift grades, young patients that engage in sport with rotational knee movements, those with recurvatum knee or ligamentous hyperlaxity, and cases of revision ACL reconstruction [12, 36].

In a systematic review study with meta-analysis, Xu et al. [5] concluded that combined ACL and ALL reconstruction may increase knee rotatory stability, reducing the pivot shift rate and moderately improving the patient’s clinical results. However, the effect of this combined ACL and ALL reconstruction on the graft rupture rate cannot be confirmed. Since they included only studies with levels of evidence I and II, Xu et al. [5] performed their meta-analysis using only six studies, which significantly reduced their number of manuscripts when compared with the present investigation. In addition, Xu et al. [5] included patients with a minimum 12-month follow-up, which we consider insufficient for this type of ACL reconstruction assessment. The criteria adopted by Xu et al. [5] generated controversy in the literature [15].

With a similar objective, Hurley et al. [13] conducted a systematic review and meta-analysis of current literature evidence to determine whether combined ACL and ALL reconstruction affects knee stability, concluding that it improves clinical results, with enhanced knee stability and lower rerupture rates. Although the authors’ [13] meta-analysis contained studies with level of evidence I, II, and III, only six articles were included because their search limit was 1 June 2019. Since then, significant clinical results have been published, corroborating the findings of these authors.

Bucar et al. [44] also used six articles in their methodology and concluded that, compared with isolated ACL reconstruction, combined ACL and ALL reconstruction did not produce significant differences in knee function. They reported that, although knee stability was slightly better in the combined ACL and ALL reconstruction group, the IKDC score and Lysholm score results were only marginally improved. Similarly to what occurred with Hurley et al. [13], the major limitation of the Bucar et al. study [44] was the literature search date (April and June 2019).

Finally, despite the good results found in this meta-analysis, there are insufficient elements to indicate routine combined ACL and ALL reconstruction. However, the present findings suggest that combined ACL and ALL reconstruction may have a beneficial role in patients at high risk of failure in isolated ACL reconstruction [12]. It is important to emphasize that more studies are needed to corroborate our results.

### Limitations

It is important to highlight some of limitations in the present study. Despite the larger sample size compared with other similar investigations, it is still considered small, which demonstrates the need for more research in the area.

Although well written, only three of the articles selected presented level of evidence I. Although this did not affect our conclusions, the larger the number of level I articles, the greater the acceptance of the scientific community as a whole.

Except for pivot shift and rerupture, most of the clinical outcomes analyzed exhibited considerable heterogeneity, according to the *I*^2^ statistic. A probable explanation would be the heterogeneity among the population of patients selected in the studies included, such as athletes or non-athletes, acute or chronic injuries, choice of graft, fixation method and surgical technique, result measures, and follow-up periods, which very likely influenced our analyses.

The explanation of the positive pivot shift test is superficial in the selected articles. This is particularly problematic, as the rotational stability potentially provided by combined ACL and ALL reconstruction is a key variable to be proven in this manuscript. As we know, pivot shift is a somewhat subjective test. Thus, we are unable to standardize how such a test was performed and measured in the studies present in this meta-analysis; thus, it could be configured as a bias. Residual pivot was considered to be any degree of postoperative pivot (I, II, or III).

Finally, another limiting factor was that some studies included patients with concomitant cartilage and meniscus injuries and the type of surgery was not clearly described, thereby potentially influencing the results obtained.

## Conclusion

Combined ACL and ALL reconstruction obtained better postoperative clinical outcomes when compared with isolated ACL reconstruction, especially in reducing residual pivot shift and rerupture rate.

## Data Availability

All data generated or analyzed during this study are included in this published article.

## Abbreviations

ACL: Anterior cruciate ligament
ALL: Anterolateral ligament
RR: Risk ratio
MD: Mean difference
CI: Confidence level
LOE: Level of evidence
NOS: Newcastle–Ottawa Scale
